# The molecular landscape of the normal human breast – defining normal

**DOI:** 10.1186/bcr3680

**Published:** 2014-06-20

**Authors:** Heidi N Hilton, J Dinny Graham

**Affiliations:** 1 Sydney Medical School - Westmead, University of Sydney at Westmead Millennium Institute, 176 Hawkesbury Road, Westmead, New South Wales 2145, Australia

## Abstract

A key approach in understanding how breast cancer can occur is to determine the regulatory pathways at play in the normal breast and to identify precisely the normal developmental mechanisms subverted during early breast cancer progression. Using normal human breast tissue samples, Pardo and colleagues have identified the gene targets and pathways displaying fluctuating expression as a consequence of the menstrual cycle. Detailed characterization of how the human breast functions in its normal state, and how this may be perturbed at its earliest point, will provide a critical step toward the prevention of breast cancer.

## 

Although breast cancer mortality rates have decreased over recent years, incidence rates continue to increase. There is broad acceptance that strategies focused on disease prevention and identification of early malignant change are most likely to contribute to real improvements in breast cancer incidence. Understanding the molecular landscape of the normal breast is a critical step in developing such approaches. The article by Pardo and colleagues [1] in the previous issue of *Breast Cancer Research* contributes an exciting new resource that addresses that goal.

Recent advances in whole-genome sequencing and expression profiling have profoundly expanded our understanding of the malignant landscape [2,3], yet the combined complement of genes expressed in cancer tissue at the time of sampling fail to provide insight into biological pathways involved in carcinogenesis. Defining the molecular events in the normal breast which contribute to the formation of a breast tumor remains elusive, and the prevention of breast cancer at its earliest stages continues to be a holy grail in breast cancer management. Although it has long been known that the hormonal milieu of the breast influences normal tissue development [4] and that exposure to exogenous hormone analogues in hormone replacement therapy and oral contraceptives contributes adversely to breast cancer risk [5,6], there is a paucity of information on the molecular events underlying this influence in humans.

Understanding regulatory pathways at play in the normal breast and the precise identification of normal developmental mechanisms subverted during early breast cancer progression are key steps toward prevention. Owing partly to a lack of appropriate models, much of our current understanding of normal breast development derives from studies in animals and, remarkably, details of how the normal human breast functions remain largely unknown. A landmark study by Longacre and Bartow [7] in 1986 described the morphological changes occurring in the normal breast during the menstrual cycle. In that study, histologic features, including mitoses, were described in a cohort of 75 premenopausal normal breast autopsy specimens, matched exactly to menstrual cycle phase by endometrial dating. The investigators discovered a striking correlation between menstrual cycle phase and proliferation, with the number of mitoses peaking with higher levels of circulating progesterone. This study was the first to directly implicate progesterone in driving cyclical development in the normal breast and is consistent with key epidemiological findings reported by Pike and colleagues [8], who postulated that this increased mitotic activity during the luteal phase is associated with a transient increase in breast cancer risk.

The article by Pardo and colleagues [1] takes the approach of Longacre and Bartow to the molecular level, resulting in an interesting series of discoveries, which both confirm and extend the findings of the 1986 study. A major strength of this new study is the utilization of normal breast tissue samples from 20 premenopausal donors to the Susan G. Komen for the Cure Tissue Bank at the IU Simon Cancer Center (KTB). By laser capture microdissecting the ductal epithelium of these samples followed by next-generation RNA sequencing, they identified the gene targets and pathways displaying fluctuating expression during the menstrual cycle in the normal human breast.

They demonstrated that 255 genes were differentially expressed between the follicular and luteal phases of the menstrual cycle. The vast majority of these (87%) displayed higher expression during the luteal phase, when serum concentrations of progesterone are higher. Significantly upregulated targets during the luteal phase were enriched for functions relating to cell cycle, cellular organization and repair, and DNA replication, recombination, and repair. These findings confirm the proliferative role of progesterone demonstrated in models of normal human breast [9] and in the murine mammary gland [10]. Data derived from mouse models have shown that progesterone regulates the mammary stem cell compartment during pregnancy and the reproductive cycle [11,12], a mechanism suggested to account for the increased window of susceptibility to transformation at these times.

Interestingly, many of the proliferative targets that Pardo and colleagues identified to be upregulated during the luteal phase are also associated with overexpression in breast cancer. Could dysregulation of these particular gene sets similarly represent a transient period of increased susceptibility to tumorigenesis during the luteal phase in the human breast? As this study shows that the genetic signature of the breast epithelium of women taking hormonal contraception resembles that of a continuous luteal phase, the altered regulation of these gene sets could also provide a potential mechanism to account for the increased breast cancer risk associated with progestin-containing oral contraception [6]. Moreover, progesterone regulation of these gene sets may underlie its hypothesized role as a promoter of malignant lesions in the breast [13-15].

As healthy women are frequently exposed to changes in their hormonal milieu, it will now be critical to determine whether this is indeed the case. Identification of such specific gene sets and pathways which contribute to increased breast tumor susceptibility could then provide novel indicators of breast cancer risk, prognosis, or treatment options (or a combination of these) and will be valuable in the development of new safer progestational agents. Moreover, as we now know that it is likely that only a subset of epithelial cells in the breast possess the potential to give rise to malignancies, these gene sets may represent key tools in the identification and isolation of such subpopulations.

Through the altruistic tissue donations by healthy women to the KTB, this unique repository of normal breast tissue and matched serum, plasma, and DNA is the largest of its kind in the world. The challenges in studying cancer initiation and progression in humans *in vivo* present a critical imperative for normal human tissue resources, such as the KTB, to facilitate investigation into the complete evolution of breast cancer and, indeed, any type of cancer. That is, in order to determine what is abnormal, we need to fully define what is normal.

## Abbreviations

KTB: Susan G. Komen for the Cure Tissue Bank at the IU Simon Cancer Center.

## Competing interests

The authors declare that they have no competing interests.

## References

[B1] Pardo I Lillemoe HA Blosser RJ Choi M Sauder CA Doxey DK Mathieson T Hancock BA Baptiste D Atale R Hickenbotham M Zhu J Glasscock J Storniolo AM Zheng F Doerge R Liu Y Badve S Radovich M Clare SE Next-generation transcriptome sequencing of the premenopausal breast epithelium using specimens from a normal human breast tissue bank Breast Cancer Res 2014 16 R26 10.1186/bcr3627 24636070 PMC4053088

[B2] Curtis C Shah SP Chin SF Turashvili G Rueda OM Dunning MJ Speed D Lynch AG Samarajiwa S Yuan Y Gräf S Ha G Haffari G Bashashati A Russell R McKinney S Langerød A Green A Provenzano E Wishart G Pinder S Watson P Markowetz F Murphy L Ellis I Purushotham A Børresen-Dale AL Brenton JD Tavaré S METABRIC Group The genomic and transcriptomic architecture of 2,000 breast tumours reveals novel subgroups Nature 2012 486 346 352 22522925 10.1038/nature10983 PMC3440846

[B3] Stephens PJ Tarpey PS Davies H Van Loo P Greenman C Wedge DC Nik-Zainal S Martin S Varela I Bignell GR Yates LR Papaemmanuil E Beare D Butler A Cheverton A Gamble J Hinton J Jia M Jayakumar A Jones D Latimer C Lau KW McLaren S McBride DJ Menzies A Mudie L Raine K Rad R Chapman MS Teague J The landscape of cancer genes and mutational processes in breast cancer Nature 2012 486 400 404 22722201 10.1038/nature11017 PMC3428862

[B4] Anderson E Clarke RB Steroid receptors and cell cycle in normal mammary epithelium J Mammary Gland Biol Neoplasia 2004 9 3 13 15082914 10.1023/B:JOMG.0000023584.01750.16

[B5] Chlebowski RT Manson JE Anderson GL Cauley JA Aragaki AK Stefanick ML Lane DS Johnson KC Wactawski-Wende J Chen C Qi L Yasmeen S Newcomb PA Prentice RL Estrogen plus progestin and breast cancer incidence and mortality in the women’s health initiative observational study J Natl Cancer Inst 2013 105 526 535 10.1093/jnci/djt043 23543779 PMC3691942

[B6] Hunter DJ Colditz GA Hankinson SE Malspeis S Spiegelman D Chen W Stampfer MJ Willett WC Oral contraceptive use and breast cancer: a prospective study of young women Cancer Epidemiol Biomarkers Prev 2010 19 2496 2502 10.1158/1055-9965.EPI-10-0747 20802021 PMC3055790

[B7] Longacre TA Bartow SA A correlative morphologic study of human breast and endometrium in the menstrual cycle Am J Surg Pathol 1986 10 382 393 10.1097/00000478-198606000-00003 3717495

[B8] Pike MC Spicer DV Dahmoush L Press MF Estrogens, progestogens, normal breast cell proliferation, and breast cancer risk Epidemiol Rev 1993 15 17 35 8405201 10.1093/oxfordjournals.epirev.a036102

[B9] Graham JD Mote PA Salagame U van Dijk JH Balleine RL Huschtscha LI Reddel RR Clarke CL DNA replication licensing and progenitor numbers are increased by progesterone in normal human breast Endocrinology 2009 150 3318 3326 10.1210/en.2008-1630 19342456 PMC2703536

[B10] Lydon JP DeMayo FJ Funk CR Mani SK Hughes AR Montgomery CAJ Shyamala G Conneely OM O’Malley BW Mice lacking progesterone receptor exhibit pleiotropic reproductive abnormalities Genes Dev 1995 9 2266 2278 10.1101/gad.9.18.2266 7557380

[B11] Asselin-Labat ML Vaillant F Sheridan JM Pal B Wu D Simpson ER Yasuda H Smyth GK Martin TJ Lindeman GJ Visvader JE Control of mammary stem cell function by steroid hormone signalling Nature 2010 465 798 802 10.1038/nature09027 20383121

[B12] Joshi PA Jackson HW Beristain AG Di Grappa MA Mote P Clarke C Stingl J Waterhouse PD Khokha R Progesterone induces adult mammary stem cell expansion Nature 2010 465 803 807 10.1038/nature09091 20445538

[B13] Colditz G Decline in breast cancer incidence due to removal of promoter: combination estrogen plus progestin Breast Cancer Res 2007 9 108 10.1186/bcr1736 17666116 PMC2206710

[B14] Santen R Yue W Heitjan D Occult breast tumor reservoir: biological properties and clinical significance Horm Canc 2013 4 195 207 10.1007/s12672-013-0145-y PMC10358155 23632998

[B15] Brisken C Progesterone signalling in breast cancer: a neglected hormone coming into the limelight Nat Rev Cancer 2013 13 385 396 10.1038/nrc3518 23702927

